# Molecular identification of *Raillietina echinobothrida* (Davaineidae) from domestic chickens in the Fergana Valley, Uzbekistan

**DOI:** 10.1590/S1984-29612026042

**Published:** 2026-09-25

**Authors:** Islomjon Zokirov, Bakhtiyorjon Abduvaliev, Mirzakarim Yunusov

**Affiliations:** 1 Fergana State University – FSU, Faculty of Life Sciences, Department of Zoology and General Biology, Fergana, Uzbekistan

**Keywords:** Cestoda, ribosomal DNA, ITS-2 sequencing, phylogenetic analysis, Gallus gallus domesticus, poultry helminths, Cestoda, DNA ribossômico, sequenciamento de ITS-2, análise filogenética, Gallus gallus domesticus, helmintos de aves

## Abstract

*Raillietina* species are among the most common cestodes of backyard chickens, but their morphological similarity complicates reliable species-level identification. This study aimed to identify *Raillietina echinobothrida* from domestic chickens (*Gallus gallus domesticus*) in the Fergana Valley, Uzbekistan, combining morphological examination with molecular analysis of the internal transcribed spacer 2 (ITS-2). Between 2021 and 2024, 80 chickens from rural households in Vodil, Sultonobod and Mirzaobod were examined by complete helminthological dissection. Recovered cestodes were assessed morphologically, and three specimens were subjected to PCR and Sanger sequencing of the ITS region (GenBank accessions PQ360975, PZ581203 and PZ581202); the ITS-2 fragment was used for comparison. *Raillietina* specimens were detected in 25.0% (20/80; 95% CI: 16.8-35.5) of the chickens (mean intensity 8.3 ± 4.0 worms per infected bird; range 2-15). Morphological characters agreed with published descriptions of *R. echinobothrida*. BLAST analysis of the 385 bp ITS-2 fragment showed the highest similarity to *R. echinobothrida* from Iraq (98.44%) and Thailand (97.39%), and maximum likelihood analysis grouped the Fergana specimens with reference sequences of this species. These findings confirm the assignment of the Uzbek material to *R. echinobothrida* and support combining morphological and ITS-2-based approaches for reliable identification of *Raillietina* species.

## Resumo

*Raillietina* spp. estão entre os cestoides mais comuns em galinhas domésticas, mas sua semelhança morfológica dificulta a identificação específica confiável. Objetivou-se identificar *Raillietina echinobothrida* de galinhas domésticas (*Gallus gallus domesticus*) no Vale de Fergana, Uzbequistão, combinando exame morfológico e análise molecular da região ITS-2. Entre 2021 e 2024, 80 galinhas de domicílios rurais em Vodil, Sultonobod e Mirzaobod foram submetidas à dissecção helmintológica completa. Os cestoides recuperados foram avaliados morfologicamente, e três espécimes foram submetidos à PCR e ao sequenciamento de Sanger da região ITS (acessos GenBank PQ360975, PZ581203 e PZ581202); o fragmento ITS-2 foi utilizado para comparação. Espécimes de *Raillietina* foram detectados em 25,0% (20/80; IC 95%: 16,8-35,5) das galinhas (intensidade média 8,3 ± 4,0 vermes por ave infectada; variação 2-15). As características morfológicas concordaram com descrições publicadas de *R. echinobothrida*. A análise BLAST do fragmento ITS-2 de 385 pb mostrou maior similaridade com *R. echinobothrida* do Iraque (98,44%) e da Tailândia (97,39%), e a árvore de máxima verossimilhança agrupou os espécimes de Fergana com sequências de referência dessa espécie. Esses achados confirmam a atribuição do material uzbeque a *R. echinobothrida* e reforçam a combinação de abordagens morfológicas e moleculares (ITS-2) para a identificação confiável de *Raillietina* spp.

## Introduction

Poultry farming, particularly within small-scale and household farming conditions, represents an important sector that contributes to food security and household income. One of the key factors adversely affecting the sustainability and productivity of this sector is gastrointestinal helminthosis (Wong et al., 2017; Shifaw et al., 2021). These parasitic infections are associated with reduced growth rate, poorer feed conversion efficiency and decreased egg production in birds. In addition to their direct effects, helminth infections may increase the severity of concurrent diseases by compromising the health and resilience of infected birds (Skallerup et al., 2005; Permin et al., 2006; Tarbiat et al., 2020).

*Raillietina echinobothrida* (Megnin, 1881) can induce nodular intestinal lesions, enteritis and granulomatous changes in the intestinal wall of domestic chickens, especially under heavy infestation (Nadakal et al., 1973; Samad et al., 1986). The species is widely distributed in tropical and subtropical regions and has been frequently reported in free-range and semi-free-range poultry (Abd El-Ghany, 2022). The involvement of ants and other arthropods as intermediate hosts allows the life cycle of this cestode to be efficiently maintained under backyard conditions: the cysticercoid stage develops within ants, and chickens become infected by ingesting parasitised ants (Nadakal et al., 1971; Malatji et al., 2025).

Species-level identification of *R. echinobothrida* has traditionally relied on light-microscopic examination of a defined set of diagnostic morphological characters, namely the subspherical armed scolex, the number and arrangement of the rostellar hooks (a double crown of hammer-shaped hooks), the shape and number of the suckers, the unilateral position of the genital pore, and the number of eggs per egg capsule in gravid proglottids (Lalchhandama, 2009; Mu et al., 2009; Butboonchoo et al., 2016). However, discriminating certain *Raillietina* species that occur in domestic chickens — notably *R. echinobothrida* and *R. tetragona* — based on morphological characters alone is not always straightforward (Butboonchoo et al., 2016). Both species possess an armed scolex, a unilaterally situated genital pore and multiple eggs per capsule in gravid proglottids; their differentiation therefore relies on whether the rostellar hooks are arranged in one or two rows, on the nearly circular versus oval shape of the suckers, and on the position of the genital pore in the anterior or posterior third of the segment (Al-Quraishy et al., 2019).

For this reason, integrating morphological analysis with molecular markers is widely regarded as a reliable strategy in modern parasitology, particularly for distinguishing morphologically similar or cryptic species (Nadler & Pérez-Ponce de León, 2011). Among cestodes, the internal transcribed spacer 2 (ITS-2) region of ribosomal DNA is one of the most informative markers for species-level identification and has been extensively used to discriminate closely related taxa and infer phylogenetic analyses (Gasser & Chilton, 1995; Ramnath et al., 2014).

Molecular studies of *Raillietina* have expanded in recent years, and the ITS-2 region has proved useful for species-level identification and phylogenetic comparison in India, Thailand and Bangladesh (Ramnath et al., 2014; Butboonchoo et al., 2016; Siddiqui et al., 2023). In Uzbekistan, however, the helminth fauna of domestic poultry has been documented mainly through faunistic and morphological surveys, in which *R. echinobothrida*, *R. tetragona* and *R. penetrans* have been recorded among the Davaineidae (Akramova et al., 2021). Molecular, ITS-based identification has only recently been applied to local helminths, as shown for *Parascaris equorum* and *Nematodirus* spp. (Turgunov et al., 2024; Sobirov et al., 2025), and integrative morphological-molecular approaches based on DNA barcoding have likewise been applied to other invertebrate taxa in the region (Zokirov et al., 2025). Consequently, molecular characterisation of *Raillietina* material from the Fergana Valley using ribosomal markers such as ITS-2 is therefore needed for the reliable species-level assignment of local isolates (Gasser & Chilton, 1995; Ramnath et al., 2014).

Despite its veterinary importance and wide geographical distribution, *R. echinobothrida* from poultry in Central Asia, and specifically in the Fergana Valley of Uzbekistan, has not been characterised molecularly, and locally generated ITS-2 reference sequences are lacking. Establishing a molecularly verified, regionally referenced record of this cestode is therefore essential for improving the differential diagnosis of morphologically similar *Raillietina* species and strengthening regional epidemiological surveillance.

The present study aimed to identify, based on morphological characters and the ITS-2 marker, specimens of *R. echinobothrida* isolated from domestic chickens reared under backyard conditions in Vodil village (Fergana district, Fergana Region) and in further localities of the Fergana Valley.

## Material and Methods

### Study area and sample collection

The study material was collected between 2021 and 2024 in rural households and small-scale farms located in Vodil village (Fergana district, Fergana Region; 40°10’31.39”N, 71°44’02.27”E; 929 m a.s.l.), Sultonobod village (Quva district; 40°33’18.20”N, 72°07’53.62”E; 494 m a.s.l.) and Mirzaobod village (Asaka district, Andijan Region; 40°40’53.10”N, 72°12’45.09’E; 481 m a.s.l.), all situated within the Fergana Valley. A total of 80 *Gallus gallus domesticus* were subjected to helminthological examination by means of standard parasitological necropsy (complete helminthological dissection) techniques: 33 birds from Vodil, 25 from Sultonobod and 22 from Mirzaobod. All birds were local indigenous (non-descript) backyard chickens and did not belong to any defined commercial breed or genetic line. Sampling was based on birds slaughtered by their owners for household consumption and was therefore non-probabilistic (convenience) in nature; no a priori calculation of the minimum sample size was performed. This constraint is taken into account when interpreting the locality-specific estimates (see Discussion). The geographic location of the sampling sites is shown in Figure 1.

**Figure 1:**
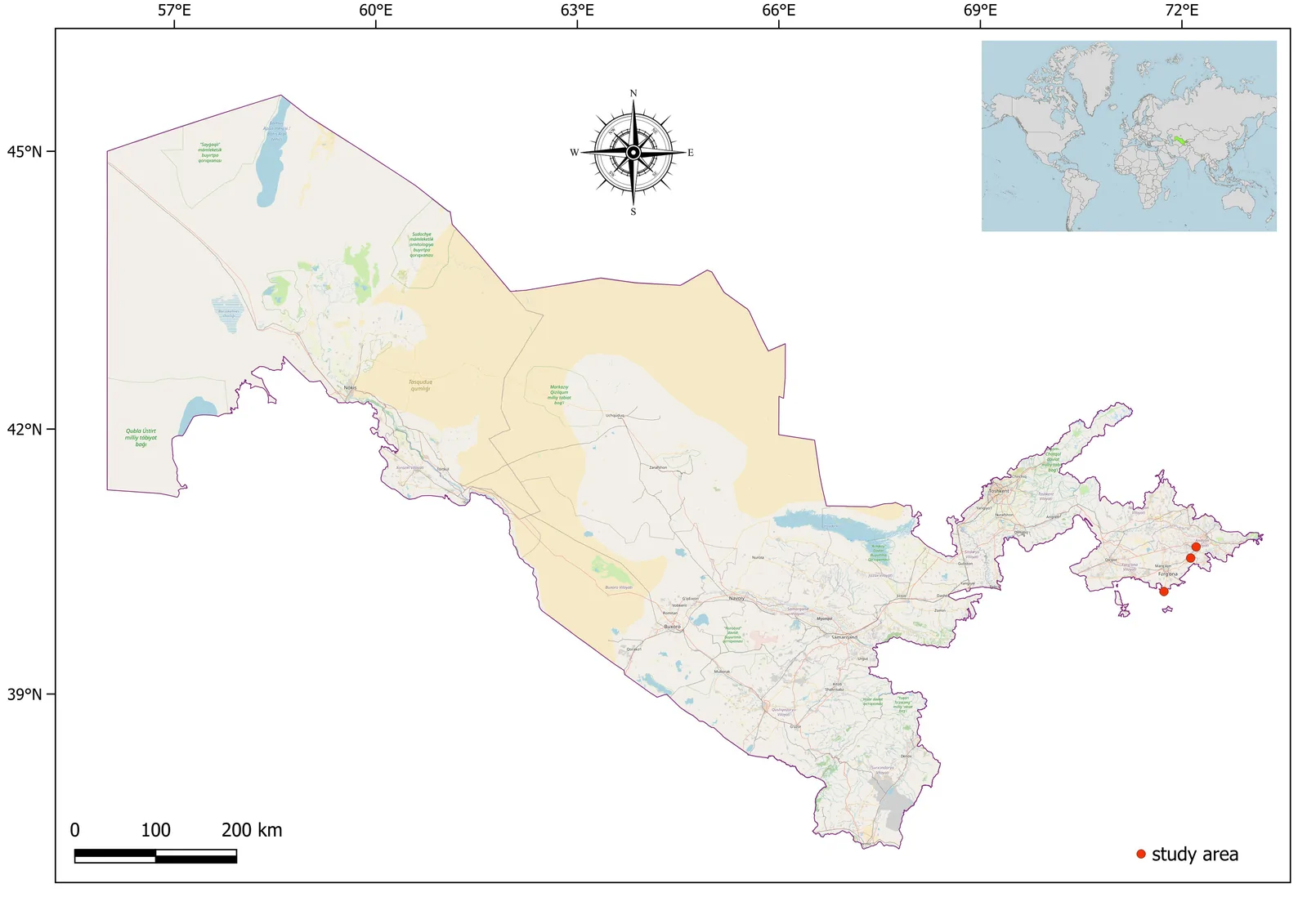
Map of the study area in the Fergana Valley (Uzbekistan), showing the three sampling localities: Vodil (Fergana district), Sultonobod (Quva district) and Mirzaobod (Asaka district).

Helminths were recovered by complete helminthological dissection, following standard procedures for the post-mortem parasitological examination of poultry (Permin & Hansen, 1998). The intestines were removed and examined separately, and recovered cestodes were washed in physiological saline and sorted for morphological and molecular study. Specimens intended for morphological analysis were fixed and stored in 70% ethanol, whereas specimens designated for molecular analysis were preserved in absolute (96%) ethanol.

The chickens included in the present study were reared under traditional backyard conditions by rural households and small-scale farms for domestic consumption. No animals were slaughtered specifically for research purposes. Slaughter was carried out by the owners as part of the routine practices of these households, without any prior intervention on the live animals by the research team. The intestines and helminthological material used in this study were obtained exclusively *post mortem*, after the routine slaughter performed by the owners for domestic consumption. Accordingly, in accordance with the FAQ guidelines of CONCEA (Question No. 45), ethical approval by an animal ethics committee (CEUA) was not required for this study.

### Morphological identification

Recovered cestodes were first sorted and examined under a stereomicroscope. Selected specimens were then stained with acetocarmine following standard procedures for cyclophyllidean cestodes (Georgiev et al., 1986; Chervy, 2024): after fixation, they were washed, stained, differentiated in acidified 70% ethanol, dehydrated through a graded ethanol series, cleared in clove oil, and mounted in Canada balsam as permanent whole-mount preparations. The preparations were examined under a light microscope to study the scolex, mature proglottids and gravid proglottids.

Recovered cestodes were initially examined based on body length, scolex structure, the presence and armament of the rostellum and hooks, the shape of the proglottids, the position of the genital pore, and the morphology of the egg capsules in gravid proglottids. A comparative approach based on previously published descriptions was used to assess the diagnostic characters typical of *R. echinobothrida* (Soulsby, 1982; Lalchhandama, 2009). Morphometric measurements were obtained from calibrated digital images captured with a light-microscope imaging system and are summarised in Table 1. Morphological identification was regarded not as a conclusion but as a preliminary step guiding the subsequent molecular analysis.

**Table 1:** Morphometric characteristics of the *Raillietina echinobothrida* specimens from the Fergana Valley (Uzbekistan).

**Morphometric character**	**Mean ± SD**
Total body length (cm)	16.83 ± 0.97
Scolex length (µm)	566.48 ± 26.53
Scolex width (µm)	580.84 ± 25.39
Sucker length (µm)	201.96 ± 9.96
Sucker width (µm)	192.34 ± 9.49
Rostellum length (µm)	69.18 ± 6.16
Rostellum width (µm)	126.60 ± 0.97

Values are given as mean ± standard deviation. Rostellar hook length (≈0.025 mm) and the number of eggs per egg capsule (6-12) are reported qualitatively in the text.

### DNA extraction and PCR

Genomic DNA was extracted from the posterior gravid proglottids of three *Raillietina* specimens using the Qiagen DNeasy Blood and Tissue Kit (Qiagen, Hilden, Germany) according to the manufacturer’s protocol. The internal transcribed spacer (ITS) region of the rDNA (comprising ITS-1, 5.8S rDNA and ITS-2) was amplified using the primers AB28 (5′-ATA TGC TTA AGT TCA GCG GGT-3′) and TW81 (5′-GGT TCC GTA GGT GAA CCT GC-3′) described by Joyce et al. (1994). PCR was performed in a final volume of 25 µL containing 10 ng of template DNA, 1 µL of each primer (10 µM), 12.5 µL of 2× Taq Master Mix (Novoprotein, Suzhou, China) and nuclease-free water. Thermal cycling consisted of an initial denaturation at 94 °C for 5 min, followed by 35 cycles of denaturation at 95 °C for 45 s, annealing at 55 °C for 45 s, extension at 72 °C for 1 min 40 s, and a final extension step at 72 °C for 5 min (Kuchboev et al., 2020).

PCR products were analysed by electrophoresis on a 2% agarose gel in a horizontal system at 100 V for 25 min, alongside a molecular-weight marker, and visualised under UV illumination (Figure 2); the amplified ITS products migrated at approximately 750 bp.

**Figure 2:**
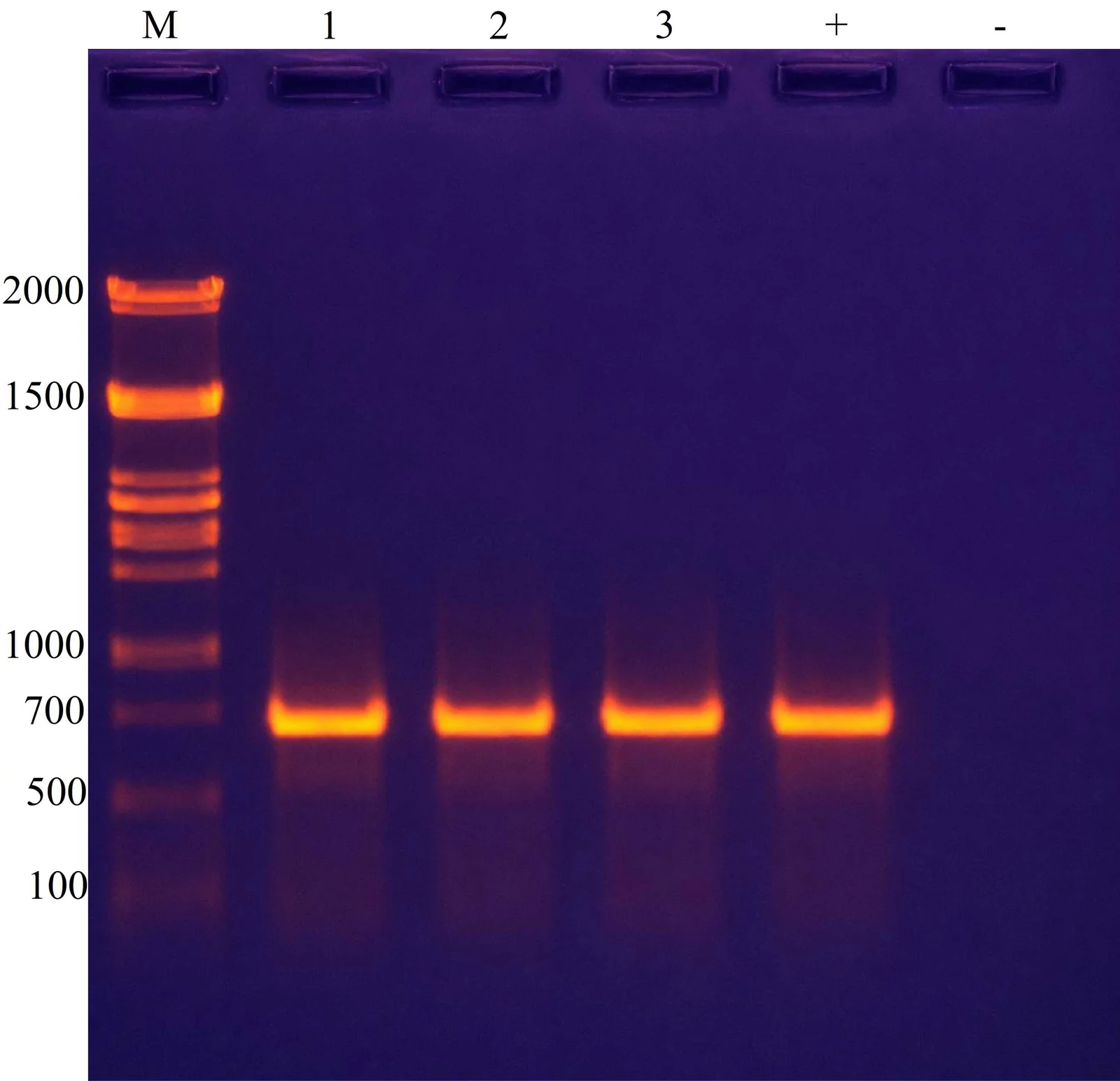
Agarose gel electrophoresis (2%) of ITS PCR products. M, molecular-weight marker; lanes 1-3, *Raillietina echinobothrida* specimens; (+), positive control (nematode DNA); (−), negative control (nuclease-free water) used to monitor potential contamination during amplification.

PCR products were submitted for Sanger sequencing to the Institute of Zoology of the Academy of Sciences of the Republic of Uzbekistan (Tashkent). The ITS-2 portion of each resulting sequence was subsequently extracted and used for downstream BLAST comparison and tree-based analysis.

### Molecular data analysis

Of the 20 *Raillietina*-positive birds, three specimens were processed for molecular analysis (Figure 2, lanes 1-3) and sequenced. The three ITS sequences were deposited in GenBank under accession numbers PQ360975, PZ581203 and PZ581202.

Twenty-five ITS-2 nucleotide sequences of species belonging to the order Cyclophyllidea were retrieved from the NCBI GenBank database and combined with the newly generated ITS-2 sequences of *Raillietina* from the Fergana Valley. *Dibothriocephalus nihonkaiensis* and *Diphyllobothrium latum* were included as outgroups. The sequences were aligned with FAMSA, and a maximum likelihood (ML) tree was inferred with VeryFastTree under the GTR+Γ substitution model with 200 bootstrap replicates.

### Statistical analysis

Prevalence was calculated as the proportion of infected birds among the total number examined and expressed as a percentage with 95% confidence intervals (CI), estimated using the Wilson score method; locality-specific prevalence was calculated separately for each study area. Infection intensity was assessed only in infected birds and expressed as the number of helminths recovered per infected host. Descriptive statistics for intensity included range, mean ± standard deviation (SD), median, and corresponding 95% CI. The normality of the infection-intensity data was assessed with the Shapiro-Wilk test. Differences in prevalence among the three localities were evaluated using the chi-square test of independence, with Fisher's (Freeman-Halton) exact test applied as a confirmatory analysis; differences in infection intensity among localities were evaluated with the non-parametric Kruskal-Wallis test. A significance level of p < 0.05 was adopted. All analyses were performed in R version 4.3.3 (R Core Team, 2024).

## Results

### Prevalence and intensity of infection

*Raillietina* specimens were recovered from 20 of the 80 domestic chickens examined in the Fergana Valley, corresponding to an overall prevalence of 25.0% (20/80; 95% CI: 16.8-35.5). The highest locality-specific prevalence was recorded in Sultonobod (Quva district), at 28.0% (7/25; 95% CI: 14.3-47.6), followed by Vodil (Fergana district) at 27.3% (9/33; 95% CI: 15.1-44.2) and Mirzaobod (Asaka district) at 18.2% (4/22; 95% CI: 7.3-38.5) (Table 2). In infected birds, the number of cestodes per host ranged from 2 to 15, with a mean intensity of 8.3 ± 4.0, a median of 8.5 and a 95% CI of 6.4-10.2; locality-specific intensity values are presented in Table 2. Prevalence did not differ significantly among the three localities (χ^2^ = 0.76, df = 2, p = 0.69; Fisher's exact test, p = 0.76). Infection-intensity values showed no significant departure from normality (Shapiro-Wilk, p > 0.05 in all localities) and did not differ significantly among localities (Kruskal-Wallis, H = 0.32, df = 2, p = 0.85). Because of the small number of infected birds per locality — in particular the four cases from Mirzaobod — these locality-specific intensity values should be interpreted with caution.

**Table 2:** Locality-specific prevalence and intensity of *Raillietina echinobothrida* infection in domestic chickens from the Fergana Valley, Uzbekistan.

**Locality**	**Examined**	**Infected**	**Prevalence % (95% CI)**	**Range**	**Mean ± SD**	**Median**	**95% CI**
Vodil (Fergana district)	33	9	27.3 (15.1-44.2)	2-15	8.0 ± 4.8	8.0	4.3-11.7
Sultonobod (Quva district)	25	7	28.0 (14.3-47.6)	3-15	8.1 ± 4.1	8.0	4.4-11.9
Mirzaobod (Asaka district)	22	4	18.2 (7.3-38.5)	6-12	9.3 ± 2.5	9.5	5.3-13.2
**Total**	**80**	**20**	**25.0 (16.8-35.5)**	**2-15**	**8.3 ± 4.0**	**8.5**	**6.4-10.2**

CI, confidence interval (Wilson score method for prevalence). Intensity is expressed as the number of helminths per infected bird. No significant differences among localities were found for prevalence (χ^2^ = 0.76, df = 2, p = 0.69; Fisher's exact test, p = 0.76) or infection intensity (Kruskal-Wallis, H = 0.32, df = 2, p = 0.85).

### Morphological observations

Recovered cestodes were whitish and ribbon-like; in fresh, fully extended material they measured 19-24 cm, whereas fixed and mounted specimens measured 16.83 ± 0.97 cm on average (Table 1). The scolex was subspherical, on average 566 µm long and 581 µm wide, and bore four muscular suckers (≈202 × 192 µm) and a rostellum (≈69 × 127 µm) armed with a double crown of small hammer-shaped hooks; detailed morphometric data are summarised in Table 1 (Figure 3).

**Figure 3:**
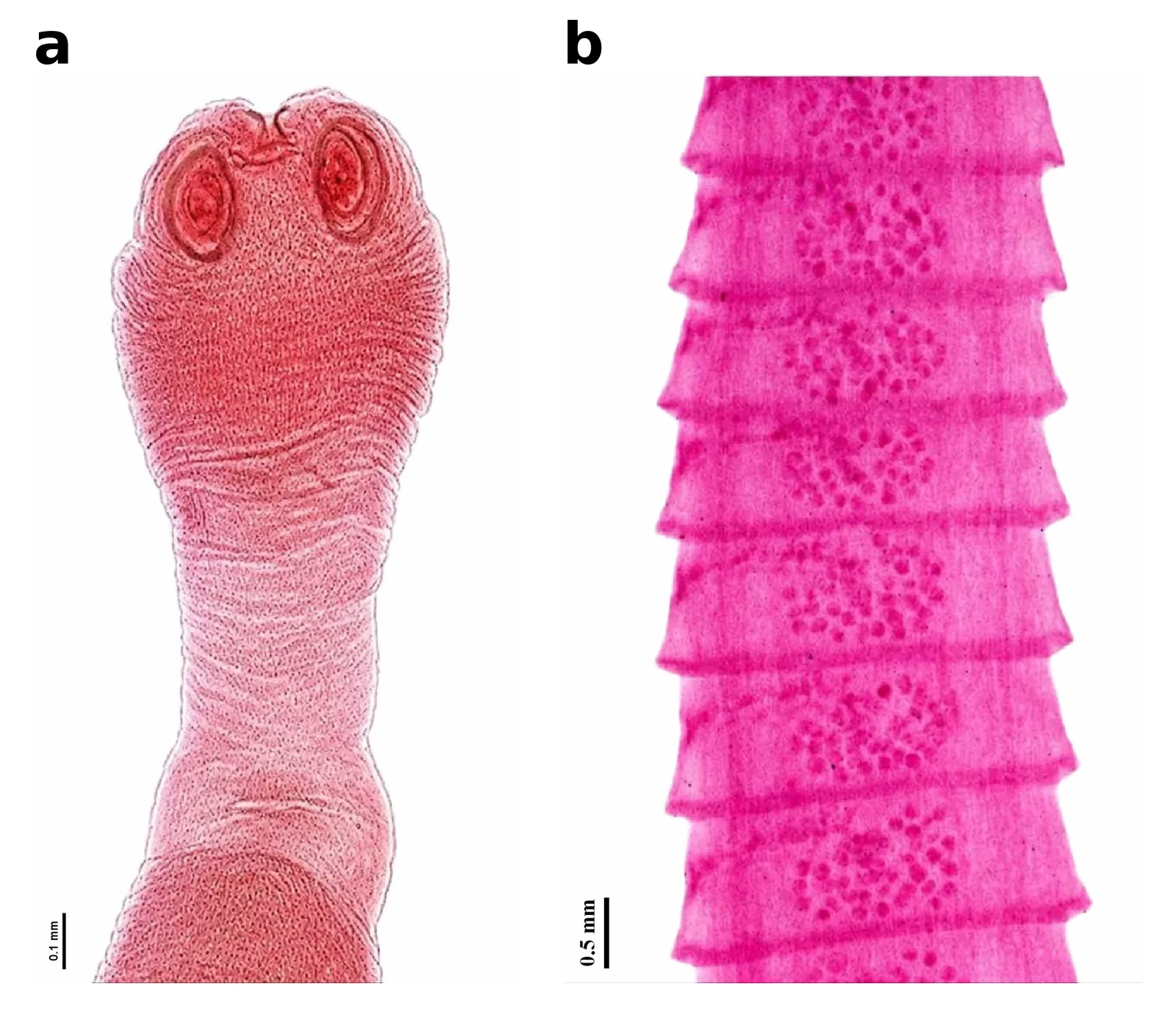
Morphological structure of *Raillietina echinobothrida*. (a) scolex bearing four suckers and an armed rostellum; (b) gravid proglottid. Scale bars are indicated on the images.

Under high magnification, the rostellar hooks were numerous and minute, measuring approximately 0.025 mm in length. Mature proglottids possessed a unilaterally situated genital pore, and gravid segments typically contained egg capsules enclosing 6-12 eggs each. These features were fully consistent with published descriptions of *R. echinobothrida*.

### Molecular results

The ITS region was successfully amplified from all three specimens, each yielding a single band of the expected size (approximately 750 bp; Figure 2). High-quality ITS sequences were obtained for the three specimens, and a 385 bp ITS-2 segment was used for comparative BLAST and tree-based analyses; the three sequences were deposited in GenBank under accession numbers PQ360975, PZ581203 and PZ581202. Chromatogram inspection revealed no ambiguous nucleotide calls or evidence of mixed-template amplification, indicating that all sequences were of high quality and suitable for downstream analyses.

BLAST analysis of the ITS-2 sequences revealed the highest similarity to an *R. echinobothrida* sequence previously reported from pigeons in Iraq (GenBank accession OQ947167). Pairwise similarity with this sequence reached 98.44%, with 379 of the 385 bp identical across the comparative fragment, corresponding to six diagnostic nucleotide differences. The second-highest similarity (97.39%) was an *R. echinobothrida* sequence from domestic chickens in Thailand (MN902343). Sequence identities with other *Raillietina* species and Davaineidae representatives were considerably lower. Collectively, these results molecularly confirmed the species-level assignment of the Fergana Valley specimens to *R. echinobothrida*.

The maximum likelihood tree based on ITS-2 sequences showed that the three Fergana sequences (PQ360975, PZ581203 and PZ581202) grouped within a well-supported clade of *R. echinobothrida* together with reference sequences from Bangladesh and Thailand (bootstrap value 100%). In the tree, *R. tetragona* was recovered as a sister species, and these two taxa formed a clade positioned adjacent to a subclade comprising *R. australis*, *R. chiltoni*, *R. dromaius* and *R. beveridgei*; *R. cesticillus* was placed on a separate branch (Figure 4).

**Figure 4:**
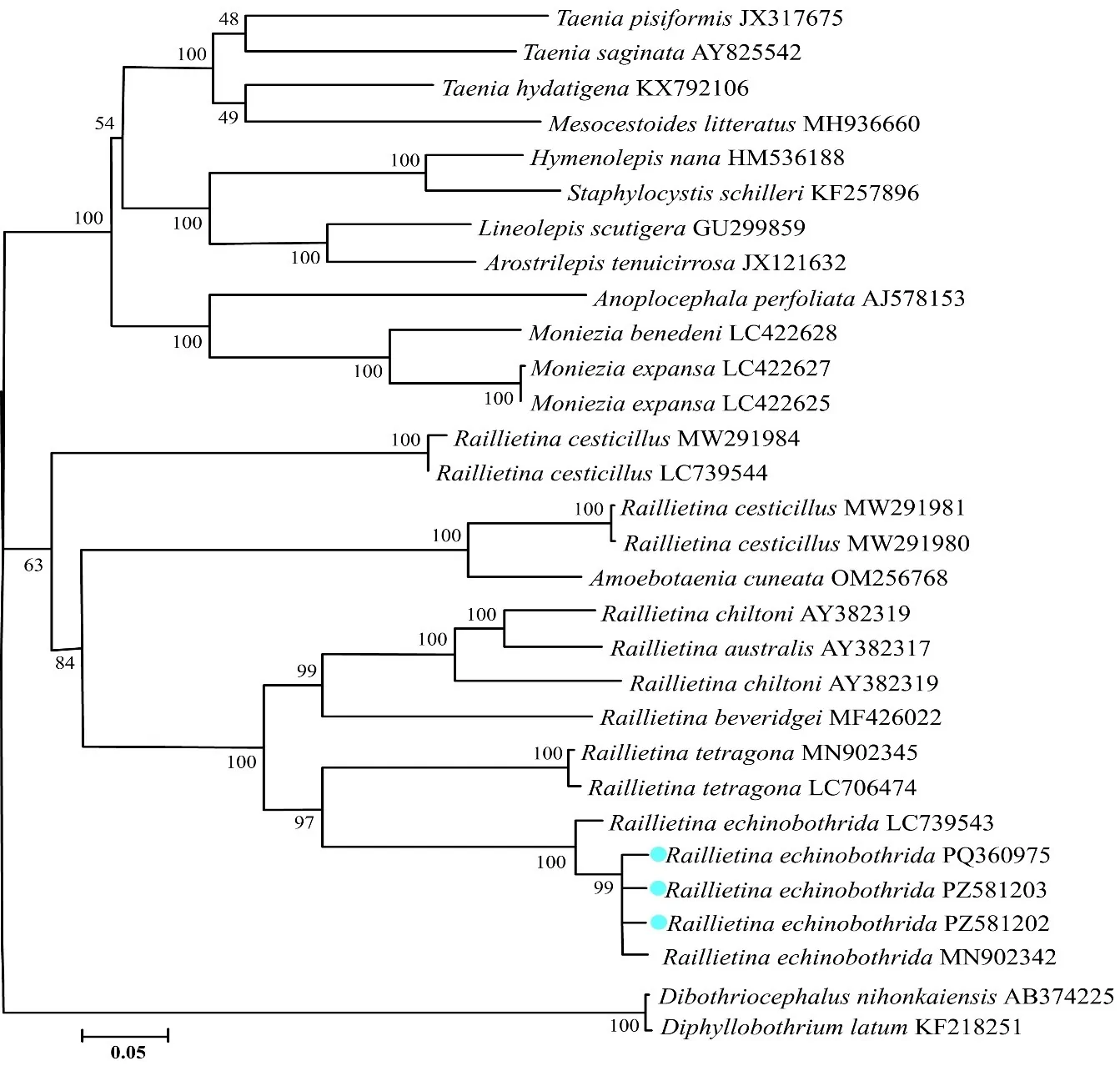
Maximum likelihood tree based on ITS-2 nucleotide sequences of *Raillietina* specimens from the Fergana Valley, Uzbekistan (blue) and related taxa retrieved from GenBank. *Dibothriocephalus nihonkaiensis* and *Diphyllobothrium latum* were used as outgroups. Numbers at the nodes indicate bootstrap support values; the scale bar indicates the number of substitutions per site.

## Discussion

The present findings demonstrate a strong congruence between morphological observations and ITS-2-based molecular analysis. The armed rostellum, the multiple rows of small hooks, the unilaterally situated genital pore and the egg capsules recorded in our specimens are all consistent with the descriptions of *R. echinobothrida* reported in the literature. Such an integrative approach is particularly important for the discrimination of morphologically similar *Raillietina* species (Lalchhandama, 2009; Butboonchoo et al., 2016).

The BLAST results further corroborated the morphological identification and confirmed the diagnostic value of the ITS-2 marker for species-level discrimination of *R. echinobothrida*. This finding is consistent with previous reports (Ramnath et al., 2014) and underscores the practical applicability of this marker to local material.

Comparative analysis of the ITS-2 sequences and the maximum likelihood tree supported the assignment of the Fergana specimens to *R. echinobothrida* and recovered *R. tetragona* as its sister species. This pattern is in agreement with the general trends reported from Thailand and Bangladesh (Butboonchoo et al., 2016; Siddiqui et al., 2023). Accordingly, the utility of the ITS-2 marker for the differentiation of morphologically similar taxa within the genus *Raillietina* is once again confirmed.

The findings also highlight the veterinary significance of *R. echinobothrida* in the Fergana Valley. The observed prevalence of 25.0% among the examined chickens indicates a substantial occurrence of the parasite in the present sample set. Direct comparison of this figure with estimates reported for other regions or studies should, however, be treated with caution, as sample size, husbandry system, seasonal conditions and host age may vary considerably between investigations. Nevertheless, *R. echinobothrida* has been consistently reported in the literature as a concern in free-range and semi-free-range chickens, which is in agreement with our observations (Abd El-Ghany, 2022). The involvement of ants and other arthropods as intermediate hosts is one of the key biological factors accounting for the persistence of this infection under backyard rearing conditions (Soulsby, 1982).

In the present study, three *Raillietina* specimens from the Fergana Valley were characterised molecularly, providing three ITS reference sequences (PQ360975, PZ581203 and PZ581202) for the region. The three sequences were mutually consistent and all clustered within the *R. echinobothrida* clade, reinforcing the species-level assignment. We note, nevertheless, that the number of sequenced isolates remains modest relative to the 20 infected birds detected, so that inferences regarding intra-population genetic variation should be regarded as preliminary; broader sampling across the three localities is planned as a next step to characterise local genetic diversity in more detail.

A further consideration is that the phylogenetic inference was based on a relatively short ITS-2 fragment (385 bp), which may limit phylogenetic resolution, particularly among closely related *Raillietina* species. Further research is therefore required to evaluate the present findings in the broader context of genetic diversity. In particular, molecular analyses based on longer sequences and additional mitochondrial markers such as COI or ND1 would enable a more in-depth investigation of intraspecific genetic variation and provide a more detailed understanding of sequence variation within the genus *Raillietina*.

Some limitations of the present study should be acknowledged. Birds were obtained by convenience from animals slaughtered by their owners for household consumption rather than by probability sampling, and no a priori sample-size calculation was performed; the locality-specific prevalence estimates therefore have relatively wide confidence intervals and should be regarded as indicative rather than definitive. Reassuringly, however, prevalence and infection intensity did not differ significantly among the three localities (p > 0.05), indicating a broadly comparable occurrence of *R. echinobothrida* across the sampled area of the Fergana Valley.

From a practical perspective, ITS-2-based verification can be integrated into regional monitoring, differential diagnosis and epidemiological surveillance of cestode infections in domestic poultry.

## Conclusion

This study confirmed, through morphological description, BLAST comparison and ITS-2-based molecular analysis, the species-level assignment of *Raillietina* specimens isolated from domestic chickens in the Fergana Valley to *R. echinobothrida*. The results demonstrate that the combined application of morphological and molecular approaches enhances diagnostic accuracy in the identification of *Raillietina* species. Furthermore, the present work enriches the molecular database available for this species under Uzbek conditions and provides a scientific basis for future monitoring, comparative studies and broader investigations into the distribution and genetic diversity of *R. echinobothrida*.

## Data Availability

All data that support the findings of this study are available within the main text of the article.
